# Dietary outcomes within the study of novel approaches to weight gain prevention (SNAP) randomized controlled trial

**DOI:** 10.1186/s12966-019-0771-z

**Published:** 2019-01-31

**Authors:** Jessica Gokee LaRose, Rebecca H. Neiberg, E. Whitney Evans, Deborah F. Tate, Mark A. Espeland, Amy A. Gorin, Letitia Perdue, Karen Hatley, Cora E. Lewis, Erica Robichaud, Rena R. Wing, the Study of Novel Approaches to Weight Gain Prevention (SNAP) Research Group, Rena R. Wing, Erica Ferguson, Ana Almeida, Kristen Annis, Ryan Busha, Isabella Cassell, Eva Chen, Pamela Coward, Jose DaCruz, Caitlin Egan, Michelle Fisher, Stephanie Guerra, Susan Himes, Brittany James, Marie Kearns, Angelica McHugh, Kevin O’Leary, Kathy Palmer, Deborah Ranslow-Robles, Amanda Samuels, Kathryn Story, Kelly Strohacker, Zeely Sylvia, Jennifer Trautvetter, Jessica Unick, Kristen Whitehead, Samantha Williams, Carolyn Wunsch, Annajane Yolken, Deborah Tate, Karen E. Hatley, Candace Alick, Shelia Barnes, Loneke Blackman, Rachel Bordogna, Kimberly Cooper, Melissa Crane, Victoria Cryer, Molly Diamond, Jennifer Frank, Noel Kulik, Hannah Lerner, Megan McMullin, Kristen Polzien, Keneisha Quick, Brooke Tompkins, Brie Turner-McGrievey, Carmina Valle, Stephen Zablonski, Erin Zeigler, Cora E. Lewis, Amy Gorin, Jessica G. LaRose, Mark A. Espeland, Letitia H. Perdue, Judy L. Bahnson, Wei Lang, Cheryl Bentley, Patty Davis, Katelyn Garcia, Leah P. Griffin, Lea Harvin, Mary A. Hontz, Mark King, Kathy Lane, Rebecca H. Neiberg, Julia Robertson, Santica M. Marcovina, Jessica Hurting, Vinod Gaur, Sonia Arteaga, Catherine Loria

**Affiliations:** 1 Department of Health Behavior and Policy, Virginia Commonwealth University School of Medicine, 830 E. Main St, 4th Floor, Richmond, VA 23219 USA; 2 Department of Biostatistics and Data Science, Wake Forest School of Medicine, Winston-Salem, NC USA; 3 Weight Control and Diabetes Research Center at The Miriam Hospital, Providence, RI USA; 4 Department of Psychiatry and Human Behavior, Alpert Medical School of Brown University, Providence, RI USA; 5 Lineberger Cancer Center and Gillings School of Global Public Health, University of North Carolina at Chapel Hill, Chapel Hill, NC USA; 6 University of Connecticut, Institute for Collaboration on Health, Intervention, and Policy, Storrs, CT USA; 7 Department of Epidemiology, School of Public Health, University of Alabama at Birmingham, Birmingham, AL USA

**Keywords:** Prevention, Weight gain, Young adults, Diet

## Abstract

**Background:**

Young adults (YA) are at high-risk for unhealthy dietary behaviors and weight gain. The Study of Novel Approaches to Weight Gain Prevention (SNAP) Trial demonstrated that two self-regulation approaches were effective in reducing weight gain over 2 years compared with control. The goal of this analysis was to examine effects of intervention on dietary outcomes and the association of diet changes with weight change.

**Methods:**

Participants were 599 YA, age 18–35 years, BMI 21.0–30.0 kg/m^2^ (27.4 ± 4.4 years; 25.4 ± 2.6 kg/m^2^; 22% men; 73% non-Hispanic White), who were recruited in Providence, RI and Chapel Hill, NC and randomized to self-regulation with Small Changes (SC), self-regulation with Large Changes (LC) or Control (C). SC and LC emphasized frequent self-weighing to cue behavior changes (small daily changes vs. periodic large changes) and targeted high-risk dietary behaviors. Diet and weight were assessed at baseline, 4 months and 2 years.

**Results:**

LC and SC had greater decreases in energy intake than C at 4 months but not 2 years. LC had the greatest changes in percent calories from fat at 4 months, but differences were attenuated at 2 years. No differences in diet quality were observed. Across conditions, increased total energy consumption, fast food, meals away from home, and binge drinking, and decreased dietary quality and breakfast consumption were all associated with weight gain at 2 years.

**Conclusions:**

This study suggests the need to strengthen interventions to produce longer term changes in dietary intake and helps to identify specific behaviors associated with weight gain over time in young adults.

**Trial registration:**

Clinicaltrials.gov #NCT01183689, registered August 18, 2010.

## Introduction

Young adulthood is a transitional period marked by significant risk for weight gain. In fact, data indicate that these years are associated with the highest rates of weight gain relative to other periods in the developmental life course and that weight gained during these years is associated with increased cardiometabolic risks later in life [1–3]. Furthermore, recent findings suggest weight gained between early and middle adulthood might also be associated with increased morbidity and mortality [4]. Thus, effective weight gain prevention in this population is of paramount importance [5].

There are myriad factors that contribute to the weight gain observed during these years, and young adulthood has been identified as a particularly high-risk period for unhealthy dietary behaviors. For example, evidence suggests that eating away from home and fast food consumption are very common in young adulthood, with more than 40% of total energy intake being consumed away from home and more frequent fast food intake associated with weight gain [6, 7]. Further, extant findings demonstrate that the transition into young adulthood is marked by increased sugared beverage consumption [8], alcohol and binge drinking [9], and decreased breakfast consumption [6], all of which can contribute to weight gain during these years. In addition to their association with weight gain, diet quality and eating related behaviors might further compound future health risks for already vulnerable young adults, given evidence linking diet quality to risk for some cancers, as well as cardiovascular disease [10, 11]. Despite mounting evidence signaling that young adulthood represents a critical time for intervention to promote healthful eating habits and prevent weight gain, relatively few studies have targeted young adults for weight gain prevention and the majority have failed to achieve positive outcomes over long-term follow-up [12–16].

The Study of Novel Approaches to Weight Gain Prevention (SNAP) Trial demonstrated that self-regulation interventions encouraging daily self-weighing, comparison of weight to a target, and corrective action can prevent weight gain in young adults over an average of 3 years of follow up [16]. Details on the trial design [17] have been published previously, but in brief, participants were randomly assigned to one of three conditions: Self-regulation with Large Changes (LC), Self-regulation with Small Changes (SC), or Self-Guided Control (C). Both active interventions directly targeted high-risk dietary behaviors and patterns common in young adulthood but did so within the context of distinct approaches. LC promoted substantial changes to calorie and fat intake to produce a weight loss buffer during the initial 8 weeks of the program, whereas the SC condition promoted small, discrete dietary changes and participants were encouraged to continue making these small changes daily forever. At 2-years, both LC and SC demonstrated efficacy for preventing weight gain relative to Control but did not differ from one another [16]. Whether LC and SC were associated with differential changes in dietary intake or eating behaviors remains unknown.

To that end, the goal of this paper was to examine changes in key dietary outcomes within the SNAP Trial and explore whether improvements in these dietary behaviors were associated with more favorable weight trajectories over 2 years of follow-up. Specifically, the aims of this paper were to: 1) determine whether the 3 groups (LC, SC, Control) differed in changes in reported total energy intake, percent of calories from fat, or diet quality as measured by the Healthy Eating Index (HEI-2010); 2) determine whether there were group differences on changes on key dietary behaviors and eating patterns (e.g., meals away from home, fast food, breakfast consumption, alcohol intake); and 3) examine the relation between change in key dietary behaviors and weight change. Based on the intervention goals and targets, we hypothesized that the LC approach would produce greater reductions in total energy and fat intake during the initial 4-month intervention. Further, we hypothesized that at 2 years, both intervention groups would have lower total energy intake and fat intake, and better diet quality, compared with Control.

## Methods

### Participants and procedures

A total of 599 participants were enrolled in the SNAP Trial. Eligible participants were women and men 18–35 years of age with a body mass index (BMI) of 21–30 kg/m^2^. Participants were excluded if they had major medical comorbidities that made unsupervised exercise or weight loss medically unsafe, if they had lost >10lbs recently, or if they were pregnant or planned to become pregnant during the initial intervention. Participants had to have internet access and be English speaking. Participants were recruited across two clinical sites (Providence, RI and Chapel Hill, NC) using a multi-method strategy and a variety of channels. See previously published reports for more details on the trial inclusion criteria [16, 17] and recruitment methods [18]. Recruitment of participants occurred from 2010 to 2012 and data collection for the 2-year visit (end point for this paper) was completed in 2014. Eligible participants were randomly assigned within strata using 1:1:1 ratio to one of the intervention conditions using a variable block length randomization scheme that was programmed centrally using a secure password protected website app.

### Intervention description

Eligible participants were randomized to one of three conditions: Self-regulation with Large Changes (LC), Self-regulation with Small Changes (SC), or Control (C). Descriptions of the interventions have been published previously [17], but are briefly described here and summarized in Table 1. LC and SC participants received 10 group sessions (weekly weeks 1–8; monthly weeks 9–16). Group sessions were facilitated by masters level interventionists with backgrounds in nutrition, exercise physiology or psychology, all of whom had training and previous experience delivering behavioral weight management treatment within the context of a research protocol. Two interventionists co-facilitated each group session. Both interventions were grounded in a self-regulation framework [19, 20] wherein participants were taught to self-weigh daily and use the scale as an error detector to adjust behaviors as needed to meet their weight control goals. After the initial 4 months, participants were instructed to continue self-weighing and report their weight via a study website, text message or email. They received monthly email feedback on weight, which was linked to a color zone system and either reinforced their success, encouraged problem solving, or encouraged additional weight control strategies to reverse observed weight gains. In addition, participants were offered optional Internet-delivered 4-week refresher campaigns twice per year to reinforce concepts taught in their assigned condition.

**Table 1:** Overview of Intervention Condition

	Large Changes (LC)	Small Changes (SC)	Self-guided (C)
Contact Schedule	Weekly group sessions weeks 1–8; monthly in weeks 9–16; two 4-week online refresher campaigns and remote reporting of weight in months 5–24	Weekly group sessions weeks 1–8; monthly in weeks 9–16; two 4-week online refresher campaigns and remote reporting of weight in months 5–24	A single group session in week 1; received an overview of both approaches and told to pick and follow the one best suited to them
Intervention Framework	Self-regulation; emphasized daily self-weighing to detect small changes in weight and take corrective action as needed	Self-regulation; emphasized daily self-weighing to detect small changes in weight and take corrective action as needed	Self-regulation; emphasized daily self-weighing to detect small changes in weight and take corrective action as needed
High-risk dietary behaviors targeted	Saturated fat; fast food; sugared beverages; alcohol; breakfast	Saturated fat; fast food; sugared beverages; alcohol; breakfast	N/A
Specific strategies to prevent weight gain^a^	Create a weight loss buffer during initial 8 weeks (5-10lbs) by reducing calories by 500–1000 and increasing moderate to vigorous activity to > 250 min / wk	Reduce energy intake by ~ 100 kcal / day and increase steps by 2000 / day for entire trial	N/A

^a^Note: shaded area represents the key differences between the interventions

Participants in LC were encouraged to create a weight loss buffer of 5-10lbs during the initial program through calorie and fat goals (1200–1800 kcals, 30% calories from fat) and an increase in moderate-to-vigorous physical activity (250 min /week). If LC participants’ weight exceeded their baseline weight at any point during follow-up, they were advised to return to their calorie goal to reverse these gains. Participants assigned to SC were taught to make daily small changes to diet equal to approximately 100 kcals per day (e.g., swap a soda for a no-calorie drink or water), and to increase physical activity by adding 2000 steps per day over their baseline level. If SC participants experienced weight gains over baseline, they were taught to make additional small changes each day to reverse these gains. Participants in the Control arm attended one in-person group session, where they received general information on weight gain in young adults and an overview of both the Small and Large Changes approaches as well as publicly available websites where additional information could be obtained on each approach; they were encouraged to choose the approach that was best suited to their personal goals. All participants received quarterly newsletters throughout the trial, as well as personalized feedback on all assessment measures after each assessment visit.

### Measures

#### Height, weight and BMI

Weight and height were assessed at baseline, 4 months and 2 years by trained research staff masked to treatment assignment. Weight was measured following a 12-h fast in light street clothes and without shoes, on a calibrated scale. Height was measured using a wall-mounted stadiometer using standard procedures. Two measures of each were obtained and the average was used. Body mass index (BMI) is calculated as follows: weight in kilograms divided by height in meters squared.

#### Dietary intake

Dietary intake was assessed using the 2005 Block Food Frequency Questionnaire (Block FFQ) at baseline, 4 months and 2 years. This validated, quantitative 110-food item questionnaire [21] is designed to assess relative intake of energy, macro- and micronutrients, and food groups. For each food item on the FFQ, participants reported the frequency with which they consumed that item as well as usual portion size over the last month. Given the intervention targets, the primary variables of interest included: change in total energy intake (kcals) and change in percent calories from fat. The Block FFQ was developed using dietary data from a nationally representative sample of the U.S. population [22] and was selected for this trial because it allowed the young adult participants to complete the assessment online at their own convenience. The Block FFQ has been used in numerous previous weight loss intervention trials, including the Diabetes Prevention Program [23].

#### Diet quality

Diet quality was measured using the Health Eating Index, 2010, which measures adherence to the Dietary Guidelines for Americans, 2010, the prevailing national dietary recommendations during the period of intervention [24, 25]. The HEI-2010 total score (out of 100) was determined using data obtained from the Block FFQ. Whereas diet quality was not a primary target of the interventions, several targets overlapped with national dietary recommendations (e.g. intake of fruits, vegetables, alcohol, added sugars).

#### Eating patterns

Estimation of ounces of diet and sugar-sweetened beverages in the past 30 days were calculated from separate questionnaire items for usual number of ounces and frequency of behavior. The first categorical question concerned frequency: “In the past 30 days, how did you consume sugar-sweetened beverages?” with responses of “none or less than one per week” converted to 0, “once per week” converted to 1, “twice per week” converted to 1, “3-4 times per week” converted to 3.5, “5–6 times per week” converted to 5.6, and “everyday” converted to 7. The second question concerned amount: “On the days that you consumed sugar-sweetened beverages over the last 30 days, how much did you drink” responses of “1 can” converted to 12 oz, “1 20-oz bottle” converted to 20, “2 cans” converted to 24 oz, “Big Gulp or 3 cans” converted to 36 oz. The answers to these two items were multiplied to estimate ounces consumed over 30 days. Parallel items and process were used for diet beverages. Alcohol intake was reported as number of drinks and the estimates were derived using data from two separate questions: “During the past 30, how many days did you have at least one drink of alcohol?” and “During past 30 days, on days you drank, how many alcoholic drinks did you drink on the average?”. Participants were also asked to report the frequency of fast food consumption, breakfast consumption and other meals away from home. These behaviors were considered important to examine given previous findings linking each of these behaviors to weight gain and / or diet quality in young adults specifically, and were drawn from the common elements items used in the EARLY Trials Consortium (https://earlytrials.org) [26].

### Statistical analyses

Differences among groups in baseline dietary quality and dietary behaviors were assessed using chi-squared tests for categorical variables and analysis of variance for continuous measures. In the case of continuous dietary quality and behavior measures, medians and interquartile ranges were also examined using non-parametric Wilcoxon Rank Sum tests to determine sensitivity to asymmetric distributions. Changes in intake and diet quality among groups from baseline to month 4 and year 2 were compared using separate regression models adjusted for baseline values. Windsorized methods were used for values above 99th percentile and below 1st percentile for change in eating frequency per day, estimated ounces of sugar sweetened beverages, diet beverages, and alcoholic beverages. Comparisons among the three groups were adjusted for multiple comparisons according to Scheffé’s method. Across all groups, relationships between weight change from baseline to month 4 or year 2 and change in dietary measures were assessed in separate models adjusting for baseline weight and respective baseline dietary measure. Partial correlations adjusted for baseline dietary measure, baseline weight, and group assignment were used to evaluate the association between dietary measure and weight change across all participants (or collapsed across the three groups). Stepwise variable selection models were employed to identify dietary measures with the strongest independent relationships with weight loss at month 4 and year 2, using *p* < 0.15 as the inclusion and exclusion criteria for variable selection; *p* < .05 was upheld in determining overall significance.

## Results

### Baseline characteristics

Participants were 78% female, 73% non-Hispanic White, and averaged 27.7 ± 4.4 years. At baseline, participants had a BMI of 25.4 ± 2.6 kg/m^2^ and the majority worked full time (58%), whereas smaller subsets both worked and were in school (21%) or were full time students only (15.6%). Relationship status was relatively evenly distributed across married (31.6%), single (31.1%), and in a relationship (33.1%), with a small number of participants living with a significant other (4.3%). The majority of participants (67%) reported an annual income of < $49,000. Baseline dietary intake behaviors are described in Table 2; the three groups did not differ significantly at baseline on any of the dietary variables.

**Table 2:** Baseline Dietary Intake and Eating Behaviors

Variable	Full Sample *N* = 599	Control *N* = 202	Large Changes *N* = 197	Small Changes *N* = 200	*p*-value
Total energy intake (kcal) Mean + SD	1663 + 611.3	1667 + 590.9	1659 + 651.6	1662 + 593.2	.99
Percent calories from fat Mean + SD	35.3 + 5.6	35.3 + 5.8	35.4 + 5.7	35.3 + 5.1	.99
Diet quality HEI-2010 Total Score (out of 100) Mean + SD	67.7 + 11.0	66.8 + 11.3	68.6 + 10.8	67.6 + 10.9	0.27
Eating frequency per day Mean + SD	4.7 + 2.3	4.4 + 1.1	4.9 + 3.0	4.7 + 2.4	.11
Meals away from home per week Mean + SD	4.6 + 3.6	4.8 + 3.7	4.2 + 3.1	4.7 + 3.9	.14
Fast food consumption per week Mean + SD	1.5 + 2.3	1.6 + 2.4	1.3 + 2.0	1.6 + 2.4	.27
Breakfast frequency per week Mean + SD Daily, N (%)	5.6 + 1.8 290 (48.4%)	5.7 + 1.7 100 (49.5%)	5.7 + 1.7 93 (47.2%)	5.6 + 1.8 97 (48.5%)	.74 .90
Sugared beverages < 1 per week, N (%) > 1 per week, N (%) Est ounces past 30 days, Mean + SD	485 (81.9%) 107 (18.1%) 48.2 + 56.3	164 (83.2%) 33 (16.8%) 44.6 + 51.3	162 (82.7%) 34 (17.3%) 47.7 + 69.0	159 (79.9%) 40 (20.1%) 52.3 + 46.2	.65 .76
Diet beverages < 1 per week, N (%) > 1 per week, N (%) Est ounces past 30 days, Mean + SD	414 (71.5%) 165 (28.5%) 77.9 + 91.4	137 (69.2%) 61 (30.8%) 85.2 + 99.4	137 (71.7%) 54 (28.3%) 67.3 + 60.1	140 (73.7%) 50 (26.3%) 80.3 + 106.5	.62 .47
Alcoholic beverages Mean + SD drinks in past 30 days	17.5 + 30.4	17.3 + 23.3	18.1 + 38.1	17.1 + 27.9	.95
Binge drinking episodes Mean + SD in past 30 days	0.9 + 1.6	1.0 + 1.9	0.9 + 1.4	0.9 + 1.4	.84

### Outcomes by intervention assignment

**Fig. 1:**
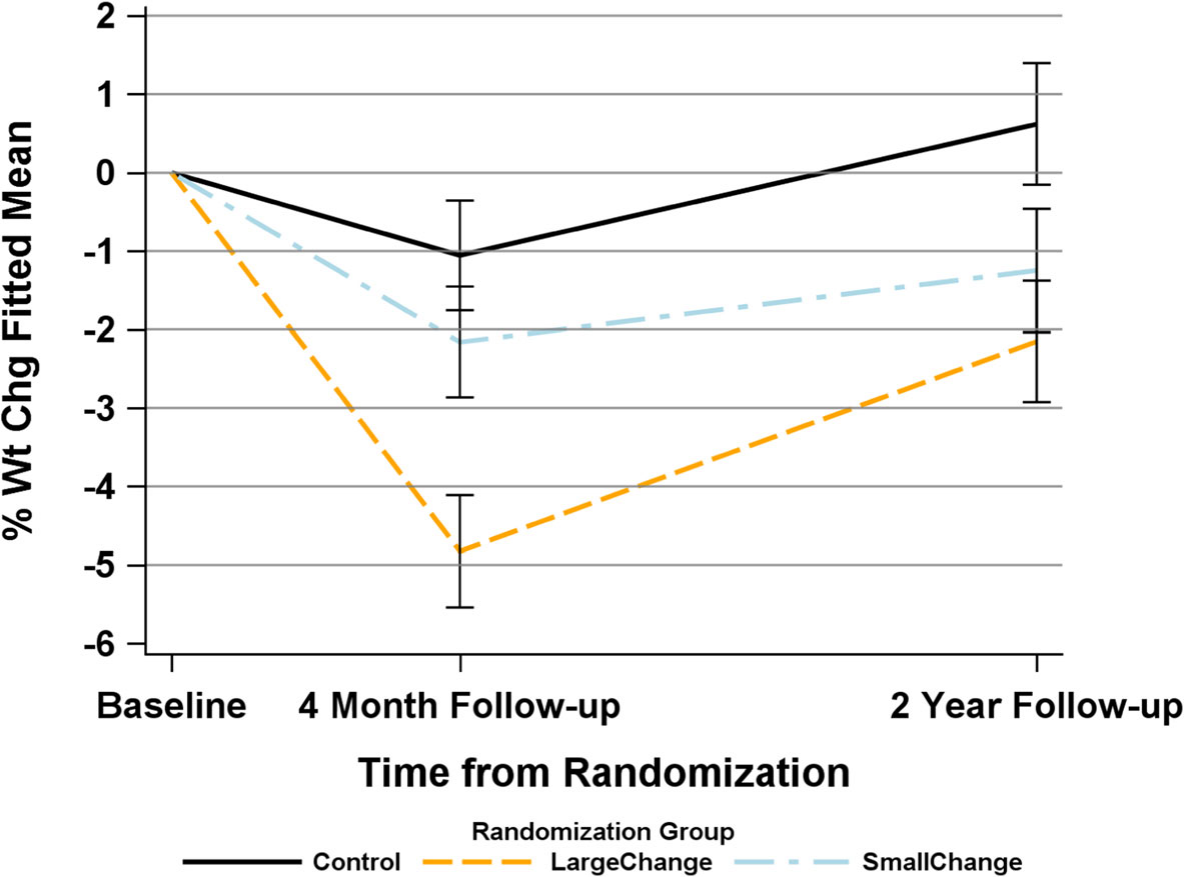
Weight Change at 4 months and 2 Years by Intervention Assignment

Weight change at 4-months and 2 years is depicted in Fig. 1. At 4-months, both intervention arms were significantly different from one another (LC vs. SC, *p* < .001) and LC was also significantly different from Control (*p* < .0001), whereas SC was not significantly different from Control (*p* = .13). At 2 years as previously reported [16] both SC and LC were significantly different from Control (*p* = .02 and p < .001, respectively) but not from one another (*p* = .33). All dietary outcomes by intervention assignment are depicted in Table 3. Changes in total energy intake differed among the three groups at 4 months, with both intervention groups reporting greater decreases in total energy than Control. At 2-year follow-up, there were no significant differences in energy intake among groups. Changes in percent calories from fat also differed among groups; at both time points LC reported greater changes in dietary fat intake than SC or C, but the differences although still significant overall were attenuated at 2 years. Although there were improvements in overall dietary quality as measured by the HEI-2010, these differences did not significantly differ among the three conditions. Additionally, at 4 months both LC and SC reported significantly greater consumption of diet beverages compared to C, but no differences persisted at 2 years.

**Table 3:** Change in Dietary Intake and Eating Behaviors at Follow-up

	Month 4				Year 2			
Dietary Behavior	Control N = 197	Large Changes *N* = 187	Small Changes *N* = 192	*P*-value	Control *N* = 178	Large Changes *N* = 174	Small Changes *N* = 172	*P*-value
Total Energy (kcal)	− 193.3 ± 26.1ǂǂ	− 275.0 ± 27.0	− 311.5 ± 26.7ǂǂ	**0.0054**	−117.2 ± 31.5	− 189.5 ± 32.0	−199.1 ± 32.2	0.14
Percent Fat	0.01 ± 0.4ǂǂǂ	−2.62 ± 0.4ǂǂǂ,ǂǂ	−0.91 ± 0.4ǂǂ	**<.0001**	1.48 ± 0.4	0.09 ± 0.4	1.58 ± 0.5	**0.0325**
Diet Quality (HEI-2010 Total Score)	1.94 ± 0.6	2.33 ± 0.6	2.61 ± 0.6	0.72	1.08 ± 0.7	1.53 ± 0.7	0.58 ± 0.7	0.62
Eating frequency per day	0.20 ± 0.17	0.14 ± 0.17	−0.01 ± 0.17	0.68	−0.24 ± 0.08	−0.20 ± 0.08	−0.24 ± 0.08	0.93
Breakfast frequency per week	0.06 ± 0.1	0.32 ± 0.1	0.31 ± 0.1	0.07	0.09 ± 0.1	0.34 ± 0.1	0.21 ± 0.1	0.26
Fast food consumption per week	−0.25 ± 0.1	−0.29 ± 0.1	−0.18 ± 0.1	0.79	0.07 ± 0.1	−0.19 ± 0.1	−0.15 ± 0.1	0.34
Meals away from home per week	−0.64 ± 0.2	−0.42 ± 0.2	−0.18 ± 0.2	0.20	0.02 ± 0.2	−0.23 ± 0.2	−0.20 ± 0.2	0.68
Sugar-sweetened beverages, Ounces per 30-day period^a^	−5.12 ± 1.37	− 5.88 ± 1.40	−6.41 ± 1.39	0.80	−5.92 ± 1.65	−2.93 ± 1.65	−2.43 ± 1.69	0.28
Diet beverages, Ounces per 30-day period^a^	−4.01 ± 2.40ǂ	5.62 ± 2.47ǂ	2.60 ± 2.51	**0.0169**	−2.99 ± 2.81	2.13 ± 2.82	− 5.90 ± 2.93	0.13
Alcoholic beverages, Drinks per 30-day period	1.33 ± 1.08	− 0.98 ± 1.07	−1.54 ± 1.07	0.14	− 0.78 ± 1.36	−1.77 ± 1.34	−0.87 ± 1.39	0.85
Binge drinking episodes, Last 30-day period	0.15 ± 0.1	0.01 ± 0.1	−0.04 ± 0.1	0.48	0.07 ± 0.1	−0.23 ± 0.1	−0.09 ± 0.1	0.28

*Note: adjustments made for baseline values of all variables. Pair-wise *p*-values reflect Scheffe adjustment for multiple comparisons. ^a^Participants reported frequency of beverage intake as part of a questionnaire on eating patterns. Ounces were estimated based on frequency of consumption and reported usual portion size (12 oz. can, 20 oz. bottle, etc.). ǂ *P* < 0.05, ǂǂP < 0.01, ǂǂǂP < 0.0001

### Dietary behaviors associated with weight change over time

Outcomes are displayed in Table 4. At both 4-months and 2 years, increases in total energy, percent calories from fat (month 4 only), fast food consumption, meals away from home, sugared beverage consumption (month 4 only), diet beverage consumption, and binge drinking were all associated with weight gain. Further, decreases in dietary quality were significantly associated with weight gain at 4 months and 2-year follow-up. Decreased frequency of breakfast consumption was associated with weight gain at the 2-year follow-up (see Table 4).

**Table 4:** Dietary Behaviors Associated with Weight Change over 2 Years

	Month 4		Year 2	
Dietary Behavior	Partial Correlation^a^	*P*-value	Partial Correlation^a^	*P*-value
Total Energy (kcal)	0.10	0.0235	0.11	0.0168
Percent Fat	0.11	0.0079	0.03	0.48
HEI-2010 Total Score	−0.15	0.0004	−0.12	0.0094
Eating frequency	−0.02	0.70	−0.03	0.46
Breakfast	−0.06	0.15	−0.14	0.0034
Fast food consumption	0.17	<.0001	0.17	0.0004
Meals away from home	0.19	<.0001	0.16	0.0006
Sugar-sweetened beverages, Ounces per 30-day period	0.13	0.0018	0.04	0.46
Diet beverages, Ounces per 30-day period	0.09	0.0315	0.02	0.74
Alcoholic beverages, Drinks per 30-day period	0.03	0.52	0.02	0.71
Binge drinking episodes, Last 30-day period	0.13	0.0047	0.13	0.0118

^a^Adjusted for Baseline Weight, Baseline Dietary Measure, and Group Assignment

To determine which of these variables had the strongest independent associations with weight changes over time, stepwise variable selection procedures were employed. These revealed that at 4-months, change in percent of calories from fat (*p* < .0001) and binge drinking (*p* = .004) were the strongest independent predictors of weight change. At year 2, change in binge drinking (*p* = .048) was the only predictor that was selected, while total calories (*p* = .07) and breakfast consumption (*p* = .059) approached significance.

## Discussion

SNAP is one of the few randomized controlled trials to report positive findings for long-term weight gain prevention in young adults [16]. The present findings further demonstrate that the interventions were associated with modest changes in dietary intake. Both LC and SC had greater decreases in total caloric intake relative to Control at 4 months. Although pairwise comparisons for changes in total energy intake were not statistically significant at 2 years, it is worth noting that both LC and SC experienced long-term reductions in the expected direction. This is consistent with our previous findings that both LC and SC were more effective in preventing weight gain at 2 years than Control, but were not significantly different from one another [16]. Further, long-term changes in dietary fat appear to favor LC, which is consistent with fat gram goals prescribed in this condition during the initial intervention. If sustained over time, it is plausible that these reductions in fat could be associated with longer-term weight outcomes, but further follow-up is needed to determine this.

Interestingly, data suggest that all groups improved diet quality modestly from baseline to month 4 and year 2 and that these changes did not differ by group. This small magnitude of changes in diet quality may be due to the fact that diet quality was relatively high in this sample at baseline. Specifically, HEI-2010 total scores averaged 67.7 out of 100 in this sample, as compared to 45.4 in a nationally representative sample of young adults [25]. Not only was diet quality quite discrepant from previous reports of young adults, but the observed baseline mean is comparable to the 95th percentile for American adults, which may reflect some self-selection bias with those who enrolled in a weight gain prevention trial being more cognizant of diet quality than the average American.

Both interventions targeted key high-risk eating behaviors that have been associated with weight gain in young adulthood – for example, decreasing fast food consumption and liquid calories, including sugared drinks (soda, coffee, energy drinks) as well as alcohol. Relative to Control, both LC and SC were associated with increases in diet beverages at 4 months, which is consistent with the core intervention materials. However, this effect was not sustained at 2 years. Further, while small improvements in other eating behaviors were observed, there were largely no group differences. The lack of long-term intervention effects for diet observed in the SNAP trial is consistent with recent findings from the CHOICES weight gain prevention trial [15] in community college students wherein some changes in eating behaviors were observed after the initial intervention relative to Control, but fast food consumption was the only dietary outcome for which effects were sustained over 2-year follow-up [27]. Given the weight differences observed in SNAP between intervention and control, it is somewhat surprising that long-term changes in these high-risk eating behaviors were not observed. This could be in part due to lower levels at baseline; as noted above, diet quality was higher than would be anticipated for a sample of young adults, and baseline fast food and sugared beverage consumption were lower than national averages, which could reflect a more health conscious sample that is not reflective of the behaviors of young adults as a whole. Of note, the lack of group differences could also be due to assessment given that we didn’t use a more fine-grained assessment such as multiple pass 24-h recalls.

Overall, increased total energy consumption, increased fast food, increased meals away from home, increased binge drinking, decreased dietary quality, and decreased breakfast consumption were associated with weight gain at 2 years. These findings are largely consistent with previous findings in cohort studies [6–9, 28] and speak to the importance of targeting these high-risk eating behaviors during these years. Of note, most intervention work targeting young adults has addressed all of these behaviors within the context of overall energy intake and more traditional weight management targets (i.e., calorie and fat goals) [29–31]. Future interventions might consider solely targeting discrete eating behaviors – for example, given the evidence linking meals away from home and fast food consumption with higher overall energy intake, poorer dietary quality, and increased risk for overweight / obesity [32], it would be of interest to determine whether targeting only these specific behaviors in the absence of a calorie goal would be sufficient to prevent weight gain and / or create an energy imbalance over time. However, given that stepwise variable selection procedures suggest that among these, binge drinking, total energy intake and breakfast had the strongest independent associations, targeting these other behaviors in the absence of overall energy goals could dampen the effects on weight control. Of note, the current findings signal that binge drinking might be an understudied dietary target linked to favorable weight trajectories over time. Given these findings and the high rates of binge eating during the transition into young adulthood [33], future work might consider developing integrated interventions to target both high-risk eating and high-risk drinking behaviors in this population.

This study is not without limitations including self-report dietary data via FFQ, which assesses long-term, usual dietary intake using a structured food list with frequency responses section [34]. It is possible that this tool was not sensitive enough to capture the differences in magnitude of total energy intake between groups over time. Although the online administration was an important pragmatic consideration with young adults, future trials targeting this age group might consider using the ASA-24 [35], which would provide a balance between rigor and practical considerations given the recalls are self-administered online. Similarly, our assessment methods likely lacked precision regarding change in key dietary variables of interest, such as quantity of sugared beverages consumed; dietary recalls would provide greater specificity for the assessment of these variables in future work. Further, because dietary intake was only assessed at baseline, 4 months and 2 years, it is unknown whether the interventions resulted in changes in diet that persisted beyond the initial intervention period, but not for a full two years. However, the attenuated and / or null results at 2 years underscore the need for continued intervention or perhaps bouts of more intensive intervention beyond what was offered in the refresher campaigns used in this trial. Finally, despite considerable targeted recruitment efforts across two clinical sites, our sample was predominantly non-Hispanic White and female, and thus, findings might not generalize to young adult men or racial / ethnic minority young adults.

Major strengths of this work include a high-risk population of young adults and a weight gain prevention trial that tested two distinct dietary approaches. This provided an opportunity to examine change in dietary variables and whether they differ by intervention assignment to further inform the discussion as to which of the intervention approaches – Large Changes or Small Changes – should be recommended widely to promote health. Given that both appear effective in preventing weight gain [16] and there do not appear to be any significant long-term differential effects on the majority of dietary outcomes, future investigations might focus on identifying baseline characteristics that can be used to predict who might be more likely to benefit from the Large Changes vs. Small Changes approach – that is, what works for whom both in terms of weight gain prevention and dietary change. Longer-term follow-up for the SNAP Trial is underway, which will help to answer these questions. Additional strengths worth noting include outstanding retention over long-term follow-up, and a generalizable sample of young adults with respect to the distribution of age, work and school status, and two clinical sites in different geographic regions of the U.S.

## Conclusions

Findings from the present study help to identify specific dietary behaviors associated with weight gain over time in young adults. Further, data point to binge drinking as a potentially novel target for future weight control interventions. And finally, taken together, the lack of sustained intervention effects observed at 2 years underscores the need for more intensive intervention approaches to produce long-term changes in diet in this high-risk population.
